# The pathology of vitamin D3 deficiency in the growing Najdi lambs

**DOI:** 10.29374/2527-2179.bjvm005223

**Published:** 2025-04-14

**Authors:** Wessam Monther Mohammed Saleh, Hayder Kamil Maryoosh Alabada, Rafid Majeed Naeem, Israa Abdulwadood Alsaad, Hussein Ali Naji

**Affiliations:** 1 Department of Internal and Preventive Medicine, College of Veterinary Medicine, University of Basrah, Basra, Iraq.; 2 Department of Livestock Services, Al-Diwaniyah Agriculture Directorate, Ministry of Agriculture, Iraq.; 3 Department of Surgery and Obstetrics, College of Veterinary Medicine, University of Basrah, Basra, Iraq.

**Keywords:** musculo-skeletal dystrophy, Najdi lambs, deficiency of vitamin D3, biochemistry, distrofia musculoesquelética, cordeiros Najdi, deficiência de vitamina D3, bioquímica

## Abstract

The current study was aimed at assessing the status of vitamin D3 in growing Najdi lambs in Basra province, Iraq. By using a model of musculoskeletal illness, “Najdi lambs”, sera were examined for determining the levels of the total serum vitamin D3, Ca (calcium), P (phosphorus), PTH (parathyroid hormone), ALP (alkaline phosphatase), and ALT (Alanine aminotransferase).The results showed that there was a sharp down-regulation (17.7 ± 1.07 Ng/ML) of total serum vitamin D3 in Najdi lambs that had signs of musculoskeletal disorders, poor body and hair condition scores, decrease appetite, and poor growth when compared to high levels (89.75 ± 7.06 Ng/ML) in control healthy Najdi lambs. However, dark coat color Najdi lambs had higher serum levels (P<0.05) of vitamin D3 than light coat color Najdi lambs in both deficient to vitamin D3 and control healthy lambs. Interestingly, a correlation between the total serum vitamin D3 concentration and the serum level of Ca/P ratio, PTH, ALP and ALT was observed. Thus in lambs with low vitamin D3 concentration, Ca and P concentrations were lower while PTH, ALP and ALT were higher than in lambs with higher concentrations of vitamin D3. In the final consideration, deficiency of vitamin D3 in local Najdi lambs potentially threatens their health and has an important role in suppressing their immunity. Our study provides evidence for association of the pigmentation and the metabolism of vitamin D3. Evaluation the serum levels of reliable minerals, hormones and enzymes as biomarkers can support the early diagnosis of metabolic diseases included vitamin D3 in domestic animals.

## Resumo

O presente estudo teve como objetivo avaliar o status da vitamina D3 em cordeiros Najdi em crescimento na província de Basra, Iraque. Utilizando um modelo de doença musculoesquelética, “cordeiros Najdi”, os soros foram analisados para determinar os níveis totais de vitamina D3 no soro, Ca (cálcio), P (fósforo), PTH (hormônio da paratireoide), ALP (fosfatase alcalina) e ALT (alanina aminotransferase). Os resultados mostraram uma redução acentuada (17,7 ± 1,07 Ng/ML) nos níveis totais de vitamina D3 no soro dos cordeiros Najdi que apresentavam sinais de distúrbios musculoesqueléticos, baixa condição corporal e capilar, diminuição do apetite e crescimento deficiente, em comparação com os níveis elevados (89,75 ± 7,06 Ng/ML) encontrados em cordeiros Najdi saudáveis do grupo controle. No entanto, cordeiros Najdi de pelagem escura apresentaram níveis séricos mais elevados (P<0,05) de vitamina D3 do que aqueles de pelagem clara, tanto no grupo com deficiência de vitamina D3 quanto no grupo controle saudável. Curiosamente, foi observada uma correlação entre a concentração total de vitamina D3 no soro e os níveis séricos da relação Ca/P, PTH, ALP e ALT. Assim, em cordeiros com baixa concentração de vitamina D3, as concentrações de Ca e P foram menores, enquanto os níveis de PTH, ALP e ALT foram mais elevados em comparação com cordeiros que apresentavam concentrações mais altas de vitamina D3. Em conclusão, a deficiência de vitamina D3 em cordeiros Najdi locais ameaça potencialmente sua saúde e desempenha um papel importante na supressão da imunidade. Nosso estudo fornece evidências da associação entre a pigmentação e o metabolismo da vitamina D3. A avaliação dos níveis séricos de minerais, hormônios e enzimas confiáveis como biomarcadores pode apoiar o diagnóstico precoce de doenças metabólicas, incluindo a deficiência de vitamina D3 em animais domésticos.

## Introduction

Cholecalciferol, or vitamin D3, is a fat-soluble vitamin involved in calcium/phosphorus hemostasis, bone formation and bone remodeling, and the development of the fetus, and it has a prospective role in protection against pathogens (Bouillon & Suda, 2014; Constable et al., 2017; Uhl, 2018; Alabada & Saleh, 2020). Likewise, the possibility of natural occurrence of CLA (caseous lymphadenitis; a disease that causes significant financial losses in the small ruminant industry) in sheep due to *Pseudomonas aeruginosa* (Saleh et al., 2019) or other pathogens is purported to be due to vitamin D3 deficiency. Insufficient sunlight exposure (mainly UVB irradiation) and/or lack of vitamin D supplement in feed are the main causes of vitamin D3 deficiency, which manifests as poor appetite and growth and osteomalacia and osteodystrophy in advanced cases (Constable et al., 2017). Numerous non-skeletal disorders are associated with vitamin D deficiency, such as inflammatory reactions, neoplastic, cardiovascular disorders, and autoimmune diseases (Holick, 2004). Reduce productivity, poor weight gain, reduce reproductive efficiency, bending of large bones, and enlargement of joints are the most important signs of vitamin D deficiency in animals (Radostits et al., 2006). However, to date, very few studies have been performed to evaluate the pathophysiology of vitamin D3 deficiency in all livestock in Iraq. To the best of our knowledge, not a single research study has been performed to evaluate the vitamin D3 status of livestock such as sheep in Basra province, Iraq, especially the breed Najdi sheep.

“Najdi Sheep” is a breed of local sheep native to the central part of the “Najd” region of the “Arabian Peninsula” (Kingdom of Saudi Arabia). Najdi sheep are adapted to live in harsh desert environment and thought that they are fewer droughts tolerant than other species such as “Awassi” breed (Alamer & Al-hozab, 2004). Phenotypically, Najdi sheep are fat-tailed and mostly black-coated sheep with long, coarse fleece; some crossbreeds have white-colored fleece. Despite the Najdi sheep being limitedly bred in Iraq, especially in Basra and the nearest south Iraqi governorates, their population in Basra is recently growing through importation (mostly from the UAE) and through breeding the pure Najdi breeds (mostly in semi-closed barns) by the local smallholders. Unfortunately, breeding of Najdi sheep in Iraq (particularly in Basra) is facing a wide range of troubles; mostly they have difficulty adapting to the Iraqi climate, their fertility declines significantly, and it is easy for them to get endemic diseases and other disorders.

Consequently, we highlighted the use of Najdi lambs raised in Basra as a more valuable experimental model to determine the correlation of the appearance of the skeleton-muscular disorders with the status of circulatory vitamin D3 concentrations. The focus of this study is therefore to demonstrate the vitamin D3 status conjugating with estimating the levels of minerals, enzymes, and hormones of poorly growing Najdi lambs bred in Basra province, Iraq.

## Materials and methods

### Experimental design

This experiment was performed from November to December 2019 in Basra province, Iraq. One hundred “Najdi lambs,” a main breed of local sheep native to the “Najd” region of the Kingdom of Saudi Arabia, 3-12 months old, were used in the current experiment. All lambs were elected from semi-closed herds of Najdi sheep with one side-closed barn with no way for grazing. Concentrated feed, hay, and water were provided ad libitum while green grasses were added intermittently. Out of 100 lambs used in the current study, 80 of them were selected and classified as the vitamin D3-deficient group (Group A) depending on exhibition signs of skeleton-muscular disorders such as bending of large bones, joint enlargements, and lameness, as well as other signs such as loss of weight gain, rough hair coat condition score, and poor body condition score. Whereas the rest (20 lambs) of the same herds at the same age group were selected as a control group (Group B), which were clinically healthy. The current study was performed following the guidelines and the rules of “The Keeping and the Using of the Experimental Animals” that were approved by the Scientific Committee, Veterinary Medicine College, Basra University, Iraq.

### Samples collection

Blood were collected aseptically from the Jugular vein following the instructions of Jackson (2013). Jugular blood (5 ML) was collected using “18G needle syringe”, then put in plain tube without EDTA and centrifuged immediately (if applicable) at 1500 RPM for ten minutes or kept overnight at the room temperature for clotting. Then sera were collected in Eppendorf tubes and kept in refrigerator at -20 °C until the time biochemical analysis.

### Biochemical analysis

All the biochemical analysis of the current study was done in the Central Researches Unit and the Clinical Pathology Laboratory, College of Veterinary Medicine, University of Basrah, Basra Province, Iraq.

#### Estimation of Lambs Vitamin D3 Concentrations

Circulatory levels of vitamin D3 in Najdi lambs were estimated using an ELISA (Enzyme Linked Immune Sorbent Assay) test kit. The biotin double-antibody sandwich technique was used for Najdi lamb serum samples. The anti-sheep vitamin D3 ELISA test procedures were performed following the guidelines and the instructions of the manufacturer (BIOASSAY TECHNOLOGY/CHINA (Bioassay Technology Laboratory, 2023).

#### Estimation of serum PTH

Serum-PTH kit (abbot/Germany) was used to estimate the serum levels of PTH in Najdi lambs. Parathyroid Hormone in Najdi lambs was automatically estimated using “ARCHITECT System Operation/ Germany”. The principle of “ARCHITECT PTH” assay is to determine of the intact PTH in serum and plasma quantitatively using “CMIA” procedure, a flexible technique protocol (Chemiflix^®^).

#### Estimation of serum ALP

Serum-ALP kit (abbot/Germany) was used to estimate the serum levels of ALP enzyme in Najdi lambs. Serum levels of ALP enzyme in Najdi lambs was automatically estimated using “ARCHITECT System Operation/ Germany”. The assay was done by using p-NPP (p-NitroPhenyle Phosphate) to measure ALP in serum as a valuable substrate.

#### Estimation of serum ALT

Serum-ALT kit (abbot/Germany) was used to estimate the serum levels of ALT enzyme in Najdi lambs. Serum levels of ALT enzyme in Najdi lambs was automatically estimated using “ARCHITECT System Operation/ Germany”.

#### Estimation of circulatory calcium concentration

Serum-Calcium kit (abbot/Germany) was used to estimate the serum levels of calcium in Najdi lambs. Serum levels of calcium in Najdi lambs was automatically estimated using “ARCHITECT System Operation/ Germany”. Arsenazo-III method, a photometric color assay was used to determine calcium in sera of Najdi lambs.

#### Estimation of circulatory phosphorus concentration

Serum-Phosphorus kit (abbot/Germany) was used to estimate the serum levels of phosphorus in Najdi lambs. Serum levels of phosphorus in Najdi lambs was automatically estimated using “ARCHITECT System Operation/ Germany” by using a phosphomolybdate method.

### Statistical analysis

All the data that were obtained from the current study were analyzed using Microsoft® Excel software and/or applying “JMP 11, SAS Institute Inc.” statistical analysis software. The differences between the results of serum levels of total vitamin D3, PTH, ALP, ALT, and Ca and P concentrations were analyzed by using “thepaired d Student's test at (P < 0.05, P < 0.0001).

## Results

**Figure 1:**
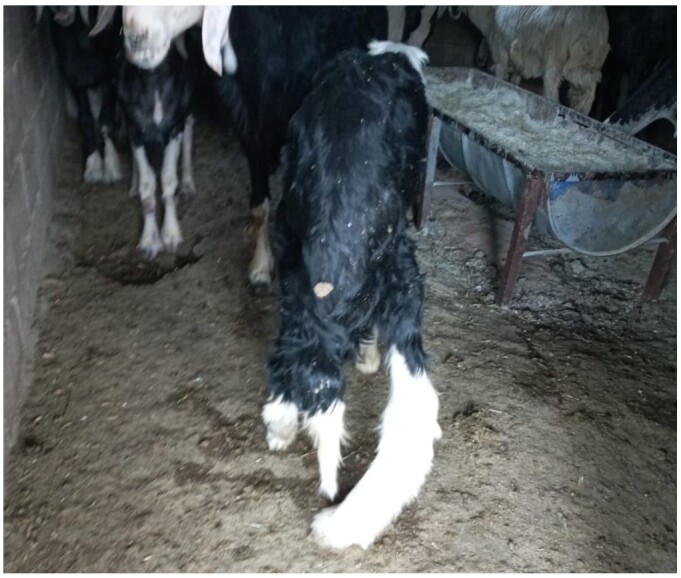
Photo of 6 months old male vitamin D3 deficient lamed Najdi lamb showing a marked bending of the right metatarsus bone combined with loss of condition.

**Figure 2:**
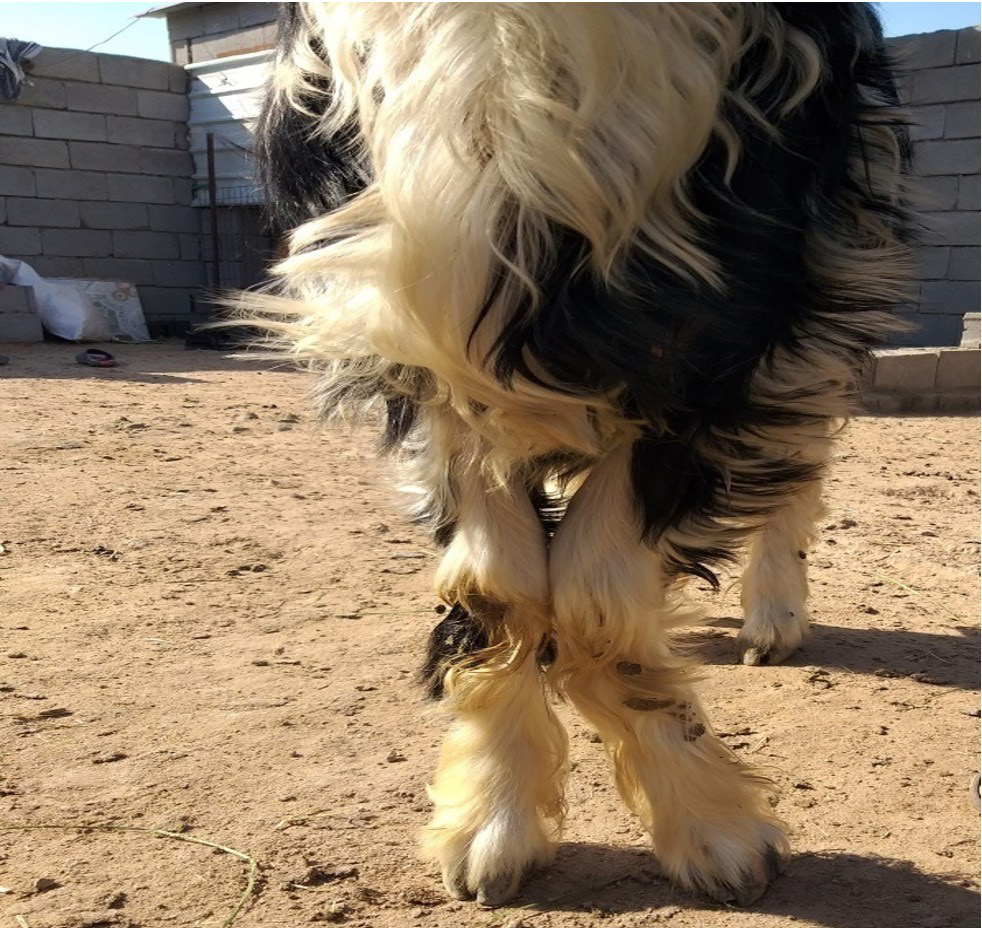
Photo of 8 months old male vitamin D3 deficient lamed Najdi lamb showing a marked bending of the left metacarpus bone combined with rough hair coat condition and poor body condition score.

A sharp (P < 0.0001) decrease (17.7 **±** 1.07 Ng/ML) of vitamin D3 concentration was observed in sera of Najdi lambs suffering from musculoskeletal disorders, poor weight gain, loss of appetite, rough hair coat condition score, and poor body condition score (Group A) compared to high levels (89.75 **±** 7.06 Ng/ML) in the control healthy Najdi lambs (Group B), as shown in (Table 1) and (Figures 1 and 2). However, coat color explained a considerable variation in vitamin D3 concentrations of dark- and light-coated Najdi lambs in both groups. Interestingly, vitamin D3 concentration was significantly (P < 0.05) dropped in the sera of white coat color Najdi lambs (in both healthy control and Najdi lambs with musculoskeletal illness) in comparison with the black coat color Najdi lambs, as shown in Table 1.

**Table 1:** Serum vitamin d3 concentrations in Najdi lambs.

**Criteria**	**Serum vitamin D3 concentration (Ng/ML), (Mean ± SE)**	
	**Group A (n=80)**	**Group B (n=20)**
Vitamin D3 concentration regardless of coat coloration	17.7 ± 1.07	89.75 ± 7.06*
Dark coat color Najdi lambs	16.76 ± 1.47^a^	91.04 ± 8.68^a^
Light coat color Najdi lambs	15.69 ± 2.29^b^	86.91 ± 13.45^b^

Group A: Lambs with Vitamin D3 Deficiency; Group B: Healthy control;

a,b Values in different superscript letters within column are significantly different at P < 0.05;

* Significantly different from the values within the same raw at P < 0.0001.

Moreover, serum calcium and phosphorus levels were markedly (P < 0.05) decreased in lambs that had lower serum levels of vitamin D3 (Table 2).

**Table 2:** Values of serum biochemical parameters of Najdi lambs.

**Criteria**	**Serum vitamin D3 concentration (Ng/ML), (Mean ± SE)**	
	**Group A (n=80)**	**Group B (n=20)**
Calcium (mg/dl)	7.26 ± 0.13 ^b^	9.42 ± 0.16 ^a^
Phosphorus (mg/dl)	3.54 ± 0.16 ^b^	5.58 ± 0.25 ^a^
PTH (pg/ml)	8.39 ± 1.72 ^a^	6.34±0.57 ^b^
ALT (U/L)	66.75 ± 5.26 ^a^	42.62 ± 2.64 ^b^
ALP (U/L)	431.46 ± 29.09 ^a^	177.00 ± 23.86 ^b^

Group A: Lambs with Vitamin D3 Deficiency; Group B: Healthy control; PTH: Parathyroid Hormone; ALT: Alanine aminotransferase; ALP: Alkaline phosphatase;

a,b Values in different superscript letters within raw are significantly different at P < 0.05.

The main result of our current study was that the lambs in Group A (which had the lowest circulatory vitamin D3 concentrations) had significantly (P < 0.05) higher circulatory levels of PTH (Table 2).

Similarly, there was an association between the declines of serum vitamin D3 concentration and the levels of ALT and ALP. Serum ALP and ALT levels were markedly (P < 0.05) high in lambs having low circulatory vitamin D3 concentrations (Table 2).

## Discussion

Of 100 Najdi lambs used in the present study, twenty of them were selected as a control group based on their good body condition score and normal vital signs. Interestingly, control Najdi lambs (Group B) found that they have high circulatory levels of vitamin D3; this result confirmed previous relevant studies conducted by Handel et al. (2016), Horst and Littledike (1982) and Nelson et al. (2016) who reported that healthy calves and lambs without skeleton-muscular disorders had high circulatory vitamin D levels. In contrast to the control healthy Najdi lambs, a sharp-decrease in the circulatory levels of vitamin D3 was observed in Najdi lambs that had various signs of skeleton-muscular disorders, poor weight gain, appetite, and poor body and hair coat condition scores (Group A). However, levels below 25 nmol/l (10 Ng/ML) are defined as actual vitamin D deficiency with negative effects on the main vitamin D functions, such as bone metabolism and calcium/phosphorus hemostasis (Norman, 2011; Weber et al., 2014). Therefore, the vitamin D3 deficient Najdi lambs of the current study did not exhibit severe signs of rickets because most of them had serum vitamin D3 levels above 10 Ng/ML. However, this study offers the first documentation of a strong correlation between the decline of serum vitamin D3 concentration and the severity of the skeletomuscular illness combined with poor body and hair condition scores that recorded Najdi lambs bred in Basra province, Iraq. Although the nutritional and the environmental conditions of the Najdi lambs in our experiment were similar, there were remarkable variations in serum vitamin D3 concentrations, which could reflect altered nutritional behavior between individuals, possible dysfunction of liver or kidneys and genetic factors. Thus, it is possible that the Najdi lambs with a declined vitamin D3 concentration were born from ewes had deficiency of vitamin D3. Similarly, a significant association between vitamin D2 and D3 in neonatal and maternal bovine plasma was reported (Constable et al., 2017), whereas low circulatory levels of vitamin D combined with poor nutrition during pregnancy might affect the vitamin D status of the fetus, this increase the risk of chronic fetal disorders later in life (Cleal et al., 2017).

Chromatically, coat color explained a considerable variation in vitamin D3 concentrations between dark and light coat color Najdi lambs in both groups. Circulatory levels of vitamin D3 were considerably (P < 0.05) down-regulated in white coat color Najdi lambs in comparison with the black coat color Najdi lambs, as shown in (Table 1). This finding suggests that the light coat color Najdi lambs have lower vitamin D3 concentrations than the dark coat color, which are probably due to the mechanism of reflecting or absorbance of the UVB irradiation. Serum vitamin D3 concentrations were higher in ewes with black heads and legs than the light coat color ewes (Zhou et al., 2019). Black sheep may have been adjusted vitamin D3 in their black coat covered areas that are adjoining with dark skin pigmentation (Mearns et al., 2008). Thus, the efficacy of the dermal conversion of 7-dehydrocholesterol to pre-vitamin D3 in dark coat color sheep may be compromised due to melanin in the pigmented skin competes with 7-dehydrocholesterol for UVB irradiation (Holick, 1981; Zhou et al., 2019). Our study provides evidence suggesting that the light coat color pigmentation contributes to the diminishing of the levels of circulating vitamin D3 contrasted to dark coat color pigmentation. However, Handel et al. (2016) have reported that the light coat color sheep had higher circulating vitamin D3 levels than the dark coat color sheep.

Circulatory vitamin D3 levels status was positively correlated in the current study with the circulating levels of Ca and P. Serum Ca and P levels were obviously (P < 0.05) depressed in lambs with the lowest vitamin D3 concentrations. Circulatory levels of Ca and P were decreased in lambs having the lowest serum vitamin D3 concentrations, whereas their levels (Ca and P) were elevated in the lambs with high circulatory levels of vitamin D3. Reduction of circulatory Ca (Demay, 2006) and P (Nisbet et al., 1966; Holick et al., 2012) may be attributed to the deficiency of vitamin D3 affecting a wide range of physiological functions. Moreover, patients with intense vitamin D3 deficiency may exhibit symptoms of hypocalcemia, whereas mild vitamin D deficiency only presents moderate declines of circulatory levels of Ca or P or may be both (Thomas & Demay, 2000).

The main interesting finding of the current study was that the Najdi lambs with the lowest circulatory vitamin D3 levels (group A) had notably (P < 0.05) elevated circulatory levels of PTH. Hormonal analysis revealed an increase in the levels of PTH in Najdi lambs that had vitamin D deficiency. Animals with rickets and osteomalacia mainly caused by vitamin D deficiency had elevated PTH (Thomas & Demay, 2000; Zongping, 2005; Craig et al., 2016). Thus, deficiency of vitamin D3 could predominantly produce hypocalcemia as approved by the current study, which thus stimulates the proliferation of PTH (Dittmer & Thompson, 2011; Craig et al., 2016; Uhl, 2018).

Similarly, there was association between the declines of serum vitamin D3 concentration and the levels of ALT and ALP. Circulatory levels of ALP and ALT were remarkably (P < 0.05) elevated in lambs having lower circulatory vitamin D3 levels, this finding suggest skeleton-muscular disorders, and probably liver disorder and kidneys dysfunction (although most reliable liver and kidney function tests were avoided in the current study). Enzymes analysis showed negative correlation between serum ALP and vitamin D3 concentrations. In the current study, ALP was considerably elevated in lambs suffering from vitamin D3 deficiency; this appears to be consistent with the results of previous relevant studies done on domesticated animals (van Saun, 2004; Radostits et al., 2007; Dittmer & Thompson, 2011; Constable et al., 2017). Elevated ALP levels appear to be caused by osteomalacia and/or osteodystrophy due to vitamin D3 deficiency in Najdi lambs, as it is associated with elevated circulatory levels of PTH, resulting in lower circulatory phosphorus levels. Our study, therefore, demonstrated that the elevations in serum ALP concentration of the Najdi lambs are not associated with rapid bone growth and remodeling observed in young growing animals (combined with higher serum phosphorus levels), but it was resulted due to vitamin D deficiency (van Saun, 2004; Dittmer & Thompson, 2011). Moreover, our study also demonstrated that vitamin D3 deficiency in Najdi lambs was associated with the elevation of serum ALT levels, because vitamin D3 deficiency can mainly lead to myopathy, thus raising circulatory ALT levels (Zongping, 2005). The elevation of ALT levels in the deficient Najdi lambs was also noticeable and it could reflect one or more illness including muscle and skeletal damages (Altug et al., 2006; Pugh & Baird, 2012; Amanoel et al., 2016; Uyar et al., 2017), acute and chronic liver disorders (Hrkovic-Porobija et al., 2017; Porobija et al., 2018), and liver flukes infestation (Denizhan et al., 2019; Khan et al., 2019). In the current study, there is evidence that vitamin D3 deficiency in Najdi lambs can be attributed to liver involvement due to liver diseases or liver flukes infestation. Moreover, liver or kidney dysfunction due to long term administration of NSAID such as paracetamol (Majeed et al., 2013) is not be excluded. However, the dysfunction of liver cells due liver damage could mainly interfere with converting pre-vitamin D to 25-hydroxycholicalcefirol leading to down-regulate production of vitamin D3 in Najdi lambs, that explain why the reliable commercial vitamin D3 therapy is not be useful in these conditions (Saleh et al., 2020).

## Conclusions

It can be concluded that the deficiency of vitamin D3 was closely involved with Ca and P down-regulation in Najdi lambs that induced various manifestations of musculoskeletal and non-musculoskeletal disorders in those deficient Najdi lambs. Hypocalcemia due to deficiency of the active form of vitamin D3 has markedly upregulated PTH production in Najdi lambs, which could induce further physiological disorders. However, we observed a relationship between the coat color and the concentration of vitamin D3 in Najdi lambs, which provides evidence for the association of the pigmentation and the metabolism of vitamin D3. Finally, liver impairment due to liver fluke infestation and/or diseases was probably one of the causes of vitamin D3 deficiency in Najdi lambs.
